# Supplementary material for: Hemophagocytic Lymphohistiocytosis Associated with Immunological Checkpoint Inhibitors: A Pharmacovigilance Study
Source: J Clin Med. 2023 Mar 2;12(5):1985. doi: 10.3390/jcm12051985 (PMC10004618; doi:10.3390/jcm12051985)
Supplement: Supplementary file 1 [file jcm-12-01985-s001.zip › jcm-2211756-supplementary.pdf]

Supplementary Table S1: Review of HLH cases associated with ICI treatment reported in literature.

Study design	Patients, gender	Cancer type	ICI	HLH criteria	HLH treatment	Time to onset (days)	Outcome	Reference
Case report	n=1 F/35	Melanoma	Ipilimumab/nivolumab	Splenomegaly, cytopenias hypertriglyceridemia, hemophagocytosis, elevated ferritin, and elevated soluble CD25 levels	1.5 mg/kg of methylprednisone	21	Recovered	Hantel A *et al.*, 2018
Case report	n=1 F/52	Metastatic melanoma	Ipilimumab	Fever, hepatitis, pancytopenia, disseminated intravascular Coagulation Ferritin elevated, triglyceride elevated	Methylprednisolone, Etoposide	56	Fatal outcome	Michot JM *et al.*, 2018
Case report	n=1, M/58	Metastatic melanoma	Pembrolizumab	Fever, anemia, thrombocytopenia, hypertriglyceridemia, hyperferritinemia reduced NK cell activity, elevated sCD163 levels	Oral prednisone	31	Recovered from HLH, permanently stopped pembrolizumab	Sadaat M *et al.*, 2018
Case report	n=1 F/36	Melanoma	Ipilimumab/nivolumab	Fever, increased soluble IL-2R (CD25), hepatitis, activation of resident von Kupffer macrophages, pancytopenia, hyperferritinemia hypofibrinogenemia	Prednisone, mycophenolate mofetil	NR >84	Recovered	Satzger I *et al.*, 2018
Case report	n=1 F/69	Metastatic melanoma	Ipilimumab/nivolumab	Fever, hepatosplenomegaly, anemia, thrombocytopenia, hyperferritinemia, hypofibrinogenemia, hypertriglyceridemia, hemophagocytosis, elevated soluble CD25, reduced functional NK cell activity	Methylprednisolone, prednisolone	NR	Recovered *TSO : four cycle of Ipi/nivo and continued fortnightly on nivolumab*	Chin CK *et al.*, 2019
Pharmacovigilance study	n=26, 77% M	Melanoma 58% NSCLC 11% Renal cell carcinoma 8% Other/unknown 23%	Anti-CTA-4 50% Anti-CTLA-4+anti-PD-1 8% Anti-PD-1 38% Anti-PD-L1 4%	Not reported	Not reported	26 (5–116)	46% recovered 23% fatal outcome 31% not reported	Davis EJ *et al.*, 2019
Pharmacovigilance study	n=38 24% F 76% M	Melanoma 55% Lung cancer 13% Bladder cancer 8% Other 24%	Nivolumab 37% Ipilimumab 18% Pembrolizumab 18% Ipilimumab+nivolumab 13%	haematological and coagulation features 15%		46.9 (median)	Recovered 61% Fatal outcome 26%	Noseda R *et al.*, 2019
Pharmacovigilance study	n=5 M/54	Pulmonary sarcomatoid carcinoma	Pembrolizumab	Fever, thrombopenia, anemia, hyperferritinaemia	Prednisolone	7	Partial control of HLH, dead from cancer progression Hscore 188	Dupré A *et al.*, 2020
	F/35	Melanoma	Ipilimumab/nivolumab	Pancytopenia, hyperferritinemia, hypertriglyceridemia, hemophagocytosis	Corticosteroids etoposide, tocilizumab	21	Partial control of HLH. Rechallenge Hscore 221	
	F/52	Melanoma	Ipilimumab/nivolumab	Fever, pancytopenia, hypofibrinogenemia, hyperferritinemia, hypertriglyceridemia, hemophagocytosis	Prednisone, etoposide	28	Death from HLH with cerebral hemorrhage Hscore 263	
	M/69	Melanoma			Corticotherapy	35		

	M/27	Melanoma	Ipilimumab<br><br>Ipilimumab/nivolumab	Fever, leukopenia, thrombopenia, hyperferritinemia<br><br>Fever, anemia, hyperferritinemia, hemophagocytosis	Methylprednisolone	28	Recovered. Rechallenge + Hscore 178<br>Recovered Hscore 247	
Case report	n=1 M/61	Head and neck squamous cell carcinoma	Pembrolizumab	Fever, pancytopenia, hepatomegaly, elevated liver enzymes, splenomegaly, hyperferritinemia, hypofibrinogenia, hypertriglyceridemia, hemophagocytosis, high soluble interleukin-2 receptor	Dexamethasone 10 mg/m2 Etoposide 150 mg/m2	298	Recovered	Kalmuk J *et al.*, 2020
Case report	n=1 F/69	Melanoma	Ipilimumab/nivolumab	Fever, liver dysfunction, anemia, thrombocytopenia, hyperferritinemia, hypofibrinogenemia, hepatosplenomegaly, hemophagocytosis	Prednisolone 1 mg/kg	42	Unknown	Mizuta H *et al.*, 2020
Case reports	n=3, M/42	Melanoma	Ipilimumab/nivolumab	Fever, hyperferritinemia, hypofibrinogenemia, hypertriglyceridemia, pancytopenia, elevated liver enzymes, hemophagocytosis	Tocilizumab	51	Recovered HScore : 319	Özdemir BC *et al.*, 2020
	M/36	Melanoma	Nivolumab	Fever, hyperferritinemia hypertriglyceridemia, pancytopenia, splenomegaly, elevated liver enzymes	Tocilizumab	78	Recovered Hscore 288	
	M/32	Melanoma	Ipilimumab/nivolumab	Fever, hyperferritinemia, hypofibrinogenemia, hypertriglyceridemia, pancytopenia, elevated liver enzymes, hemophagocytosis	Tocilizumab	91	Recovered Hscore 317	
Case report	n=1 M/54	Pulmonary metastasis of renal cell carcinoma	Ipilimumab/nivolumab + carbozantinib	Fever, anemia, thrombopenia, elevated liver enzymes, hyperferritinemia, hypofibrinogenemia, hemophagocytosis	Methylprednisolone, anakinra	NR	Recovered Hscore 202 *TTO : 6 days after his second infusion of nivolumab/ipilimumab with cabozantinib*	Azari A *et al.*, 2021
Case report	n=1 M 33	Melanoma	Ipilimumab/nivolumab	Fever, elevated liver enzymes thrombopenia, hyperbilirubinemia, hepatomegaly, splenomegaly, hyperferritinemia, hypertriglyceridemia	Prednisone 1mg/kg increased 2mg/kg on day 2	NR	Recovered *Diagnosed with encephalitis and hepatitis associated with HLH* *TTO* : Post 2 cycles of Ipi/nivo	Ghous G *et al.*, 2021
Pharmacovigilance study (FAERS Database)	n=199 M 119 (59.7%) F 69 (34.6%) UK 11 (5.5%) Median age 65	Melanoma (69, 34.6%), Non-small cell lung cancer (61, 30.6%) Renal cell carcinoma (12, 6%).	Pembrolizumab (60, 30.2%) Nivolumab (54, 27.1%) Ipilimumab/nivolumab (47, 23.6%)	NR	NR	NR	Death 53 (26.6%) Critical 36 (18.1%) Hospitalization 74 (37.2%) Unknown 36 (18.1%)	Grewal US et al., 2021

Multi-center study	n=2<br>M/69<br>M/56	NR<br>NR	Nivolumab<br>Ipilimumab/nivolumab	NR<br>NR	Corticotherapy<br>Corticotherapy	252<br>168	Recovered<br>Recovered	Kramer R *et al.*, 2021
Case report	n=2<br>M/75	Lung cancer	Pembrolizumab	Fever, cytopenia, elevated liver enzymes, hyperferritinemia, hemophagocytosis	Corticotherapy	46	Hscore 175<br>Recovering	Kurozumi A *et al.*, 2021
	F/60	Lung cancer	Pembrolizumab	Cytopenia, coagulation abnormalities, elevated liver enzymes, hyperferritinemia	Corticotherapy	30	Hscore 185<br>Recovering (improved her laboratory finding)	
Case report	n=1<br>M/68	Metastatic renal cell	Nivolumab/ipilimumab	Fever, splenomegaly, pancytopenia, hyperferritinemia, hypertriglyceridemia hypofibrinogenemia, high CD25, low NK cell activity	Dexamethasone	90	Recovered<br>Hscore 233	Masood A *et al.*, 2021
Case report	n=1<br>F	Melanoma	Nivolumab/ipilimumab	NA	Corticotherapy		Recovered<br>Positive rechallenge	Holmes Z *et al.*, 2022<br>•
Case report	n=1<br>F/80	Squamous cell carcinoma	Pembrolizumab	Pancytopenia, hepatitis, fever, splenomegaly, hyperferritinemia, hypofibrinogenemia, hypertriglyceridemia, elevated soluble interleukin (IL)-2 receptor, low NK-cell activity	Corticotherapy tocilizumab and etoposide	180	Recovered	Marar R *et al.*, 2022
Case report	n=1<br>M/67	Lung adenocarcinoma	Atezolizumab	Fever, hepatomegaly, splenomegaly, pancytopenia, hyperferritinemia, hyperbilirrubinemia, hypertransaminitis, elevated IL-6 and CD25, hemophagocytosis	Dexamethasone, tocilizumab, anakinra, mycophenolate mofetil and etoposide	14	Death, neurological dysfunction<br>Hscore 256	Rubio-Perez J *et al.*, 2022
Case-report	n=2<br>F/50	Thymic carcinoma	Pembrolizumab	Fever, splenomegaly, elevated liver enzymes, thrombocytopenia, anemia hypertriglyceridemia, hypofibrinogenemia, hyperferritinemia, decreased natural killer (NK) and elevated soluble CD25	Dexamethasone, etoposide	7	Recovered	Wei Y *et al.*, 2022
	M/70	No small cell lung cancer	Pembrolizumab	Fever, splenomegaly, elevated liver enzymes, hyperferritinemia, elevated soluble CD25, hypertriglyceridemia, hemophagocytosis,	Dexamethasone, etoposide	7	Recovered<br>EBV+	
Pharmaco vigilance study	n=190<br>36% M	Melanoma 40%<br>Lung cancer 22.1%<br>Leukemia 3.7%<br>Renal cancer 5.3%<br>Bladder cancer 2.6%	Nivolumab 21.6%<br>Pembrolizumab 30.5%<br>Ipilimumab/nivolumab 34.2%	NA	NA	102 (median, n=50)	Recovered 32.6%<br>Fatal outcome 15.3%	Our series
